# Quality of life and well-being during the COVID-19 pandemic: associations with loneliness and social isolation in a cross-sectional, online survey of 2,207 community-dwelling older Canadians

**DOI:** 10.1186/s12877-023-04350-x

**Published:** 2023-09-30

**Authors:** Jennifer Briere, Sophia Haotong Wang, Ulfat Ara Khanam, Josh Lawson, Donna Goodridge

**Affiliations:** 1 Department of Psychology and Health Studies, University of Saskatchewan, Saskatoon, Canada; 2 Health Sciences Program, College of Medicine, Canadian Centre for Health and Safety in Agriculture, Respiratory Research Centre, University of Saskatchewan, Saskatoon, Canada; 3 Department of Medicine and Canadian Centre for Health and Safety in Agriculture, College of Medicine, University of Saskatchewan, Saskatoon, Canada; 4 College of Medicine, Respiratory Research Centre, University of Saskatchewan, Saskatoon, Canada

**Keywords:** Quality of life, Well-being, Loneliness, Social isolation, Older adults, COVID-19 pandemic

## Abstract

**Background:**

The far-reaching health and social sequelae of the COVID-19 pandemic among older adults have the potential to negatively impact both quality of life (QoL) and well-being, in part because of increased risks of loneliness and social isolation. The aim of this study was to examine predictors of QoL and well-being among Canadian older adults within the context of the pandemic, including loneliness and social isolation.

**Methods:**

This cross-sectional, online survey recruited older adult participants through community organizations and research participant panels. Measures included the: Older People’s Quality of Life Scale-B, WHO-5, DeJong Gierveld Loneliness Scale, Lubben Social Network Scale and five COVID-19 specific items assessing impact on loneliness and social isolation. Multiple linear regression models were used to adjust for potential confounders.

**Results:**

A total of 2,207 older Canadians (55.7% female, with a mean age of 69.4 years) responded to the survey. Over one-third strongly disagreed that the pandemic had had a significant effect on either their mental (35.0%) or physical health (37.6%). Different patterns of predictors were apparent for QoL and well-being. After adjusting for all variables in the models, the ability of income to meet needs emerged as the strongest predictor of higher QoL, but was not associated with well-being, except for those who chose not to disclose their income adequacy. Age was not associated with either QoL or well-being. Females were more likely to experience lower well-being (β=-2.0, 95% C.I. =-4.0,-0.03), but not QoL. Reporting three or more chronic health conditions and that the COVID-19 pandemic had a negative impact on mental health was associated with lower QoL and well-being. Loneliness was a predictor of reduced QoL (β=-1.4, 95% C.I. =--1.6, -1.2) and poor well-being (β=-3.7, 95% C.I. =-4.3,-3.0). A weak association was noted between QoL and social isolation.

**Conclusions:**

The COVID-19 pandemic is associated with differential effects among older adults. In particular, those with limited financial resources and those with multiple chronic conditions may be at more risk to suffer adverse QoL and well-being consequences. Loneliness may be a modifiable risk factor for decreased QoL and well-being amenable to targeted interventions.

## Background

Older adults have been profoundly affected by the COVID-19 pandemic, declared in Canada in March 2020. Between March 2020 and October 2021, adults aged 65–84 accounted for an estimated 42% of the 35,000 COVID-19 deaths in Canada, while those 85 years and older accounted for 48% [1]. Age-related vulnerabilities such as chronic diseases, functional limitations, and mental health conditions have magnified pre-existing inequities in health and access to health care [2]. Older adults demonstrated consistently high levels of compliance to pandemic public health messaging that urged social distancing, limiting in-person contact, and avoiding large crowds, [3–6] potentially compromising opportunities for social engagement [7]. The high costs of food, shelter, and fuel have resulted in increasing financial pressures, particularly for older individuals on fixed incomes [1]. Each of these factors has the potential to negatively impact quality of life (QoL) and well-being of older adults.

Maintenance of good QoL for older adults has been globally endorsed as a critical focus of attention for governments and policy makers [8, 9]. QoL can be considered a dynamic, subjective, and multidimensional concept that incorporates both micro-individual and macro-societal factors, reflecting a cognitive assessment of relative satisfaction with domains important to an individual [10]]. Despite age-related increased risk of physical, cognitive, and social impairments, most older adults evaluate their QoL positively in terms of health, material circumstances, social contacts, dependency, and social comparisons [11].

In contrast to QoL, well-being refers to an individual’s emotional response to their circumstances and is reflected in the presence of positive emotions and contentment, with the absence of persistent negative emotions [12, 13]. Well-being can be described as feeling good and evaluating life positively [14]. Determinants of well-being include good health, availability of and access to basic resources such as adequate income and support, and positive social relationships [15]. The simultaneous evaluation of the complementary concepts of QoL, which involves evaluative and cognitive appraisal, and well-being, which provides insight into the quality and intensity of emotional experiences, is considered to provide a more robust assessment than either concept alone [16].

Prior to the COVID-19 pandemic, loneliness among older adults had already been a well-recognized public health concern, given its adverse effects on mental and physical health, well-being and mortality [17–19] Loneliness refers to an emotionally painful subjective feeling resulting from mismatches between the desired and actual quantity and quality of social connections.[20]. One-third of older adults will experience some degree of loneliness in later life [21]. Marked (37-67%) increases in loneliness were noted during the early months of the pandemic (April-December, 2020) compared to 2011–2015 data from the Canadian Longitudinal Study on Aging [2]. Attribution of these increases included a range of factors, such as limitations in community support and health services, reduced access to transportation, and decreased opportunities for social participation and engagement [2]. Personal and environmental factors such as personality traits, relationship expectations, physical and mental health, and cultural norms may all contribute to an individual’s perception of loneliness, regardless of the level of social contact [22]. Social isolation, on the other hand, refers to an objective state of having few or infrequent social contacts [23]. Socially isolated individuals, however, may not consider themselves lonely, [23] with several studies reporting only a weak correlation between loneliness and social isolation [24, 25].

Both loneliness and social isolation have been associated with negative health outcomes such as cardiovascular disorders, functional decline, and mental health issues [26]. There is also evidence that loneliness and social isolation are related to decreased QoL [27–31] and well-being, [32, 33] although these findings have been mixed [22, 31, 34–38]. Given the unique circumstances of the COVID-19 pandemic and its restrictions with their implications for loneliness and social isolation among older adults, the associations with QoL and well-being merit further study in order to inform both practice and policy. The aim of this study was to examine factors associated with QoL and well-being among older adults within the context of the pandemic, including loneliness and social isolation.

## Methods

This cross-sectional, online survey followed the Checklist for Reporting Results of Internet E-Surveys (CHERRIES) quality reporting guidelines [39] and received ethical approval from the University of Saskatchewan Behavioural Ethics Board (BEH #2669).

### Sample and recruitment

Respondents were recruited using three strategies. General recruitment occurred between July and October, 2021 through both local seniors’ collective housing complexes and 338 older adult-focused not-for-profit and advocacy groups across Canada. Twenty-eight organizations from all provinces, excluding Quebec, agreed to relay the advertisement regarding the study to their members and affiliates using their chosen means (e.g., email lists, newsletters, digital or virtual posters). The advertisement described the study and directed interested persons over age 55 years to either directly access the online survey or telephone the research lab to schedule telephone completion with an assistant over the phone. A total of 149 respondents were recruited through this strategy. Additionally, two research support services, the *Canadian Hub for Applied and Social Research* (CHASR) and *Qualtrics*, were contracted to facilitate recruitment of eligible respondents in their respective participant panels. Between September and November, 2021, respondents from the CHASR panel completed telephone interviews (n = 617), while 1,857 Qualtrics panelists completed an online survey in early December, 2021. As an incentive, respondents could elect to enter a draw for one of 45 $100CDN. Personal data related to entry in the draw was collected separately and not linked to the data provided.

The online survey used in general recruitment and CHASR recruitment was hosted on Survey Monkey, while Qualtrics used their own survey platform to host the survey. For all surveys from both platforms, repeated survey completion was permitted, so that multiple eligible participants sharing devices could access and take the survey. To screen for duplicate responses, participants were asked to create a personal code (consisting of the first three letters of their mother’s name, the day and month of their birth) at the beginning of the survey that would not identify them personally.

The exclusion criteria for the survey included age less than 51 years old, and residing outside of Canada. However, anyone interested could complete the survey and ineligible responders were screened out during data analysis (omitted *n =* 416). With consideration for older adults’ technological skills (e.g., accidentally exiting the survey, unable to return to the survey), incomplete survey responses were included in the analysis, with missing data described in the analysis section.

### Measures

QoL was evaluated using the 13-item Older People’s Quality of Life Scale-B (OPQoL-B) [40]. This is a brief form of the original OPQoL, [41] which contains 35 items related to both health and broader QoL domains such as health, social relationships, independence, control over life, home and neighborhood, psychological and emotional state, leisure and social activities, and financial circumstances. The original OPQoL [42] was developed using survey and qualitative data from older adults to establish social relevance to those older than 65 years of age. The OPQOL had been validated in community-dwelling older adults and ethnically diverse populations when assessed against other measures of QoL in older age, such as the CASP-19 and WHOQOL-OLD, and meets standards for reliability (Cronbach’s alpha = 0.856, with corrected item-total reliability correlations exceeding 0.30) [41]. The OPQoL-B items are scored on a scale from Strongly agree = 1to Strongly Disagree = 5 and are summed, with positive items reverse coded. The total score ranges from 13 to 65, with higher scores indicating higher QoL [40]. The OPQoL-B is considered suitable for measuring QoL outcomes in community-dwelling populations of older adults [42].

Well-being was assessed with the widely used WHO-5 scale [43]. This measure is comprised of five items scored from 0 (at no time) to 5 (at all times) and assesses positive mood (good spirits, relaxation), vitality (being active, waking up refreshed and rested), and general interest (being interested in things) over the previous two weeks [43]. The score is calculated by summing the scores for each item and multiplying by 4, with 0 indicating the worst imaginable well-being and 100 indicating the best possible well-being [43]. Scores of 50 or less indicate poor well-being [43]. The construct and clinical validity of the WHO-5 across setting and disease conditions are rated as very high [44].

The DeJong Gierveld (DjG) 6-item scale [45, 46] was used to evaluate overall loneliness with three negatively worded statements examining emotional loneliness (missing an intimate relationship), and three positively worded statements about social loneliness (missing a wider social network). For the negatively worded items, neutral and positive answers are scored as one point, while on the positively worded items, neural or negative answers are scored as one point. The range of scores range from 0 (least lonely) to 6 (most lonely). The scale can be used as a one-dimensional, cumulative score measure, [45] which was the score used in this analysis. Research using this scale demonstrates good internal consistency with coefficients ranging from 0.80 to 0.90, with excellent congruent validity [45].

Social engagement with family and friends was measured using the six item, 5-point Likert Lubben Social Network Scale (LSNS-6), [47] which is designed to gauge social isolation in older adults by assessing perceived social support. Respondents indicated the number of people (0 = None to 5 = Nine or more) in their network whom they felt could be relied upon for social support. The total score is calculated by summing all items for a total possible score of 30. Higher scores indicate more social engagement. The LSNS-6 has strong internal consistency (Cronbach α ≥ 0.83) [47].

In order to specifically examine the ways in which the COVID-19 pandemic had affected respondents’ loneliness and social networks, five items were drawn from the American Association of Retired Persons (AARP) Foundation and United Health Foundation survey on social isolation [48]. The items were measured with a 5-point Likert scale and were as follows: (a) I have lost touch with many people since the COVID-19 pandemic; (b) It takes a lot of energy to connect with friends during the COVID-19 pandemic; (c) The increased social isolation from the COVID-19 pandemic has had a significant negative impact on my mental health; (d) The increased social isolation from the COVID-19 pandemic has had a significant negative impact on my physical health; and (e) The COVID-19 pandemic has caused my stress and/or anxiety levels to increase. No psychometric data are yet available for these items.

### Data analysis

Statistical analyses were completed using the Statistical Package for the Social Sciences (SPSS) version 28 (IBM). For categorical variables, descriptive statistics were calculated using frequencies (%). For continuous variables, descriptive statistics were calculated using mean, standard deviations (S.D.), median, minimum, and maximum.

Multiple linear regression models were fitted to adjust for potential confounders for each of two outcomes: QoL and wellbeing. The strength of association was determined by beta-coefficients and 95% confidence intervals (C.I.). Initially, potential confounders were identified as variables of clinical importance based on the literature and within our data. Then, statistical significance was determined for the potential covariates in crude analysis. These confounders and covariates were fitted to the model and made up the base model. The base model was adjusted for age, sex, region, ethnicity, marital status, family members, living with roommate, number of years in the current residence, education, how often income met participants’ needs, social contacts per day, pets, number of chronic conditions, social network and isolation, and COVID related variables. After determining the base model, multiple linear regression analysis was performed to examine the association between loneliness (DjG) and QoL by adding each of these variables to the base model.

## Results

### Participant characteristics

The total number of respondents for the survey was N = 2,623; 617 respondents were recruited through CHASR whose protocol include telephone contact. A small number of participants who accessed the survey online called the laboratory telephone for assistance with the survey (approximately n = 6), but the exact number was not recorded. There were 2,207 participants included in the adjusted analyses. A total of 1,218 cases were used for the OPQoL outcome (44.8% missing) and a total of 1,242 cases were used for the WHO well-being outcome (43.7% missing) No imputations were conducted for the missing data. Total missing numbers (%) have been presented in separate columns in Tables 1 and 2.

The mean age of participants was 69.4 years (S.D.=8.2). Table 1 describes the participants’ personal, social, and health characteristics. Just over half of the respondents were female (55.7%), while 56.8% were married or in a common-law relationship and 32.1% had a bachelor’s degree or higher level of education. The majority (87.8%) of respondents claimed White ethnicity. Daily social contact with between 0 and 4 people was reported by 53.5% and 49.9% had a pet. The majority (70.4%) reported no or only one chronic health condition, with arthritis (65.1%) being the most common.

**Table 1:** Participant Characteristics

Variables	Overall n = 2207		Missing (%)
	n	column%	
Sex			7 (0.3)
Male	975	44.3	
Female	1225	55.7	
Region			10 (0.5)
British Columbia	248	11.4	
Ontario	606	27.7	
Quebec	228	10.4	
Prairies (AB, SK, MB)	975	44.6	
Atlantic (NB, NS, NL, PEI)	127	5.8	
Marital status			14 (0.6)
Single-never married	280	12.8	
Divorced/Separated	389	17.7	
Widowed	279	12.7	
Married or Common-Law	1245	56.8	
Ethnicity			28 (1.3)
White	1913	87.8	
Asian/Pacific Islander	105	4.8	
Other	161	7.4	
Family members			71 (3.2)
Live alone	713	33.4	
Live with spouse or common-law	949	44.4	
Live with family other than spouse or common-law	474	22.2	
Live with roommate			722 (32.7)
No	1409	94.9	
Yes	76	5.1	
Number of years in the current residence			40 (1.8)
2 years or less	104	4.8	
3–5 years	297	13.7	
6–10 years	350	16.2	
> 10 years	1416	65.3	
How often your income meets your needs			17 (0.8)
None of the time	204	9.3	
Some of the time	694	31.7	
All of the time	1237	56.5	
Prefer not to say (Undisclosed)	55	2.5	
Education			18 (0.8)
High school or less	631	28.8	
Post-secondary	842	38.5	
Bachelor’s degree or more	702	32.1	
Prefer not to say	14	0.6	
Social contact per day			29 (1.3)
0–4 persons	1158	53.5	
5–9 persons	606	28.0	
10–19 persons	236	10.9	
20 + persons	166	7.7	
Pet			33 (1.5)
Yes	1084	49.9	
No	1090	50.1	
Number of chronic conditions			none
None	802	36.3	
One	753	34.1	
Two	436	19.8	
Three or more	216	9.8	
**Chronic conditions**			
Arthritis			
No	1436	65.1	
Yes	771	34.9	
Cancer			
No	1987	90.0	
Yes	220	10.0	
Chronic respiratory diseases			
No	2076	94.1	
Yes	131	5.9	
Dementia			
No	2200	99.7	
Yes	7	0.3	
Diabetes			
No	1813	82.1	
Yes	394	17.9	
Heart disease			
No	2010	91.1	
Yes	197	8.9	
Stroke			
No	2141	97.0	
Yes	66	3.0	
Mood Anxiety disorder			
No	1944	88.1	
Yes	263	11.9	

Table 2 provides an overview of the results using descriptive statistics of the continuous and COVID-19 related variables, while Table 3 compares mean scores on the OPQoL, WHO-5, and DjG between age groups using ANOVA. Analysis was conducted using three age groups (51–65, 66–80 and 80 + years) following standard age divisions cited in other publications [49]. Statistically significant differences between age groups were noted, with the most positive scores on all three measures for those over 80 years of age.

**Table 2:** Descriptive statistics of the continuous and COVID-19 related variables

Variables	n	Mean	Median	SD	Min	Max	Missing (%)
Age	2164	69.4	69.0	8.2	51.0	100.0	43 (1.9)
**COVID-19 variables**							
COVID 1 - I have lost touch with many people since the COVID-19 pandemic	2151	3.0	3.0	1.4	1.0	5.0	56 (2.5)
COVID 2 - It takes a lot of energy to connect with friends during the COVID-19 pandemic	2146	3.1	3.0	1.3	1.0	5.0	61 (2.8)
COVID 3 - The increased social isolation from the COVID-19 pandemic has had a significant negative impact on my mental health	2144	2.5	2.0	1.3	1.0	5.0	63 (2.9)
COVID 4 - The increased social isolation from the COVID-19 pandemic has had a significant negative impact on my physical health	2144	2.4	2.0	1.3	1.0	5.0	63 (2.9)
COVID 5 - The COVID-19 pandemic has caused my stress and/or anxiety levels to increase	2147	2.8	3.0	1.4	1.0	5.0	60 (2.7)
**Scales**							
Social Engagement (Lubben)	2113	14.3	14.0	6.5	0.0	30.0	94 (4.3)
Loneliness (DeJong)	2132	2.5	2.0	1.9	0.0	6.0	75 (3.4)
**Outcome variables**							
Older People’s Quality of Life (OPQoL)	2075	53.7	54.0	8.2	13.0	65.0	132 (6.0)
Well-Being Index (WHO)	2126	59.7	64	24.0	0.0	100.0	81 (3.7)

SD = Standard deviation, min = minimum, max = maximum

Lubben = Lubben Social Network Scale

DJ = DeJong Gierveld Loneliness Scale

WHO = World Health Organization Well-Being Index (WHO-5)

OPQoL = Older People’s Quality of Life

**Table 3:** Comparison between Quality of Life (OPQoL), Wellbeing (WHO-5), and loneliness (DjG) by age groups

Age group	Quality of Life (OPQoL) Mean (SD)	WellBeing (WHO-5) Mean (SD)	Loneliness (DjG) Mean (SD)
51–65 years	51.7 (8.5)	56.1 (24.8)	2.8 (2.0)
66–80 years	54.5 (8.0)	60.8 (23.6)	2.4 (1.9)
> 80 years	56.2 (7.0)	67.1 (21.3)	1.7 (1.7)
P-value*	< 0.001	< 0.001	< 0.001

* Comparing differences by age group using ANOVA

### Impact of COVID-19

**Fig. 1:**
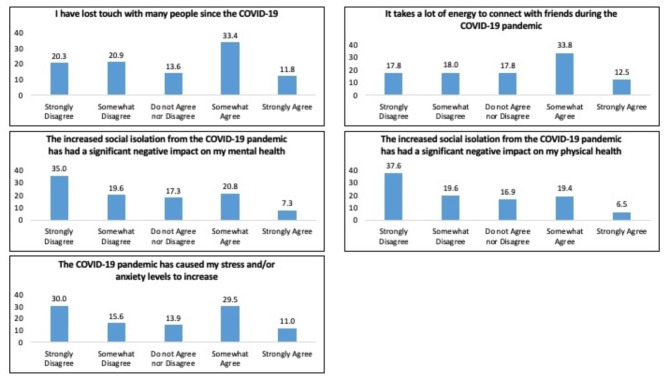
Impact of COVID-19 variables

Figure 1 displays the responses to each of the COVID-19 pandemic items on social relationships, mental and physical health and stress and/or anxiety. More than one-third of respondents strongly disagreed that the pandemic had a negative effect on either their mental health (35.0%) or physical health (37.6%).

**Fig. 2:**
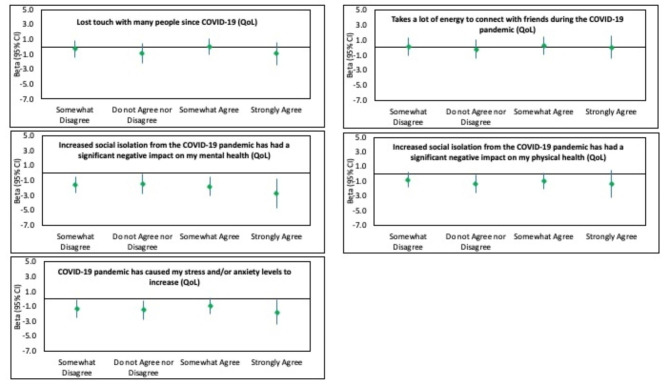
Associations between impact of COVID-19 and Quality of Life. *Adjusted for age, sex, region, marital status, ethnicity, family members, live with roommate, number of years in the current residence, how often your income meets your needs, education, social contact per day, pet, Number of chronic conditions, social network scale (Lubben), and loneliness scale (DeJong)

**Fig. 3:**
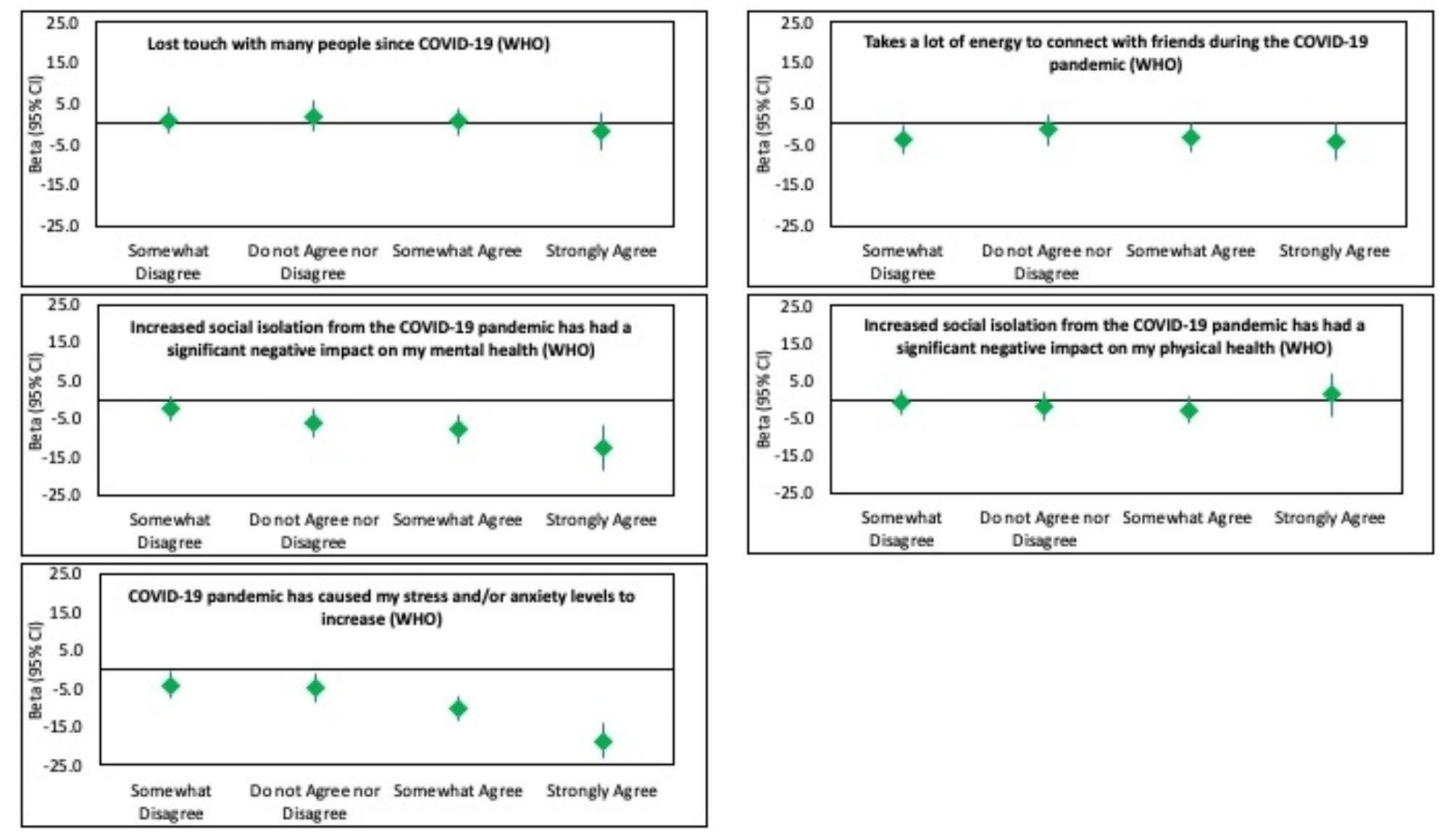
Associations between impact of COVID-19 and Wellbeing. *Adjusted for age, sex, region, marital status, ethnicity, family members, live with roommate, number of years in the current residence, how often your income meets your needs, education, social contact per day, pets, number of chronic conditions, social network scale (Lubben), and loneliness scale (DeJong)

Figure 2 illustrates the associations between the impact of COVID-19 and QoL, while Fig. 3 depicts the relationships between the impact of the pandemic and well-being. The two items relating to mental health (*“Increased social isolation from the COVID-19 pandemic has had a significant negative impact on my mental health”* and *“The COVID-19 pandemic has caused my stress and/or anxiety levels to increase”*) had stronger relationships with both QoL and well-being than losing touch with people, the energy required to connect with people or the pandemic’s effect on physical health. At least for well-being, there appeared to be dose-response relationships.

### Factors associated with quality of life and well-being

Table 4 presents the crude and adjusted analyses examining the associations between personal, social, and health characteristics, loneliness and social engagement and the outcomes of QoL and well-being. After adjusting for all variables in the model, the ability of income to meet needs emerged as the strongest predictor of higher QoL. Older adults who reported that their income always met their needs had a QOL, on average, 5 points higher than those whose incomes never met their needs. Age was not associated with QoL or well-being in the adjusted models.

**Table 4:** Crude and adjusted† analysis examining the associations with the Older People’s Quality of Life Scale and WHO-5 Wellbeing Index scales

Variables	Older People’s Quality of Life		WHO-5 Wellbeing Index	
	Crude Analysis β-coefficient 95% CI	Adjusted Model β-coefficient 95% CI	Crude Analysis β-coefficient 95% CI	Adjusted Model β-coefficient 95% CI
Age	**0.2 (0.16, 0.25)**	0.02 (-0.03, 0.1)	**0.4 (0.3, 0.6)**	-0.03 (-0.2, 0.1)
Sex (ref: Male)				
Female	0.2 (-0.6, 0.9)	0.2 (-0.5, 0.8)	**-4.5 (-6.5, -2.4)**	**-2.0 (-4.0, -0.03)**
Region (ref: British Columbia)				
Ontario	-0.3 (-1.6, 0.9)	-0.1 (-1.3, 1.0)	-0.6 (-4.2, 3.0)	-1.1 (-4.5, 2.2)
Quebec	**1.7 (0.2, 3.2)**	0.9 (-0.5, 2.3)	**8.8 (4.5, 13.2)**	**5.2 (1.0, 9.4)**
Prairies (AB, SK, MB)	**2.7 (1.6, 3.9)**	0.6 (-0.6, 1.7)	**4.5 (1.1, 7.9)**	-0.3 (-3.6, 3.0)
Atlantic (NB, NS, NL, PEI)	-1.5 (-3.3, 0.2)	-1.2 (-2.8, 0.3)	2.4 (-2.8, 7.6)	-0.4 (-5.0, 4.3)
Marital status (ref: Single-never married)				
Divorced/Separated	-1.1 (-2.4, 0.2)	-0.7 (-2.7, 1.3)	-2.3 (-6.0, 1.4)	2.1 (-3.9, 8.1)
Widowed	**2.2 (0.9, 3.6)**	0.1 (-2.3, 2.5)	3.4 (-0.7, 7.4)	2.1 (-5.0, 9.3)
Married or Common-Law	**3.2 (2.1, 4.3)**	1.2 (-0.7, 3.0)	**6.9 (3.8, 10.0)**	4.6 (-0.9, 10.1)
Ethnicity (ref: Caucasian)				
Asian/Pacific Islander	**-2.3 (-3.9, -0.6)**	**-1.9 (-3.4, -0.4)**	2.4 (-2.4, 7.2)	1.9 (-2.5, 6.3)
Other	-0.9 (-2.3, 0.5)	0.9 (-0.4, 2.2)	**4.6 (0.6, 8.6)**	**7.6 (3.6, 11.6)**
Family members (ref: Live alone)				
Live with spouse or common-law	**2.7 (1.8, 3.5)**	3.5 (-7.6, 14.5)	6.9 **(**4.5, 9.2**)**	10.1 (-23.0, 43.2)
Live with family other than spouse or common-law	-0.1 (-1.1, 0.8)	3.2 (-7.8, 14.2)	-1.2 (-4.0, 1.7)	6.1 (-27.0, 39.1)
Live with roommate (ref: Yes)				
No	**4.3 (2.3, 6.2)**	-0.6 (-2.9, 1.8)	**9.5 (4.0, 15.0)**	-3.8 (-10.8, 3.2)
Number of years in the current residence (ref: 2 years or less)				
3–5 years	1.6 (-0.3, 3.5)	1.4 (-0.5, 3.2)	2.5 (-2.9, 8.0)	4.2 (-1.3, 9.7)
6–10 years	**2.7 (0.8, 4.6)**	1.0 (-0.8, 2.8)	**6.2 (0.9, 11.6)**	4.5 (-0.9, 9.9)
10 years+	**4.7 (3.0, 6.4)**	1.5 (-0.2, 3.1)	**8.5 (3.6, 13.4)**	**5.2 (0.2, 10.1)**
How often your income meets your needs (ref: None of the time)				
Some of the time	**5.0 (3.8, 6.2)**	**2.7 (1.3, 4.0)**	**9.0 (5.4, 12.7)**	0.8 (-3.2, 4.9)
All of the time	**10.6 (9.5, 11.8)**	**5.0 (3.6, 6.3)**	**20.2 (16.7, 23.6)**	2.9 (-1.1, 7.0)
Prefer not to say (Undisclosed)	**8.9 (6.5, 11.4)**	**4.2 (1.5, 6.8)**	**21.3 (14.0, 28.6)**	**9.3 (1.5, 17.2)**
Education (ref: High school or less)				
Post-secondary	-0.1 (-0.9, 0.8)	**0.9 (0.1, 1.7)**	0.04 (-2.5, 2.6)	1.7 (-0.7, 4.0)
Bachelor’s degree or more	**1.1 (0.1, 2.0)**	**1.1 (0.2, 2.0)**	2.3 (-0.3, 5.0)	2.1 (-0.4, 4.7)
Prefer not to say	-2.5 (-7.2, 2.3)	0.9 (-4.8, 6.6)	-8.6 (-22.4, 5.1)	-8.1 (-25.2, 8.9)
Social contact per day (ref: 0–4 persons)				
5–9 persons	**2.8 (2.0, 3.7)**	0.1 (-0.6, 0.9)	**5.3 (2.9, 7.7)**	0.4 (-1.9, 2.6)
10–19 persons	**3.4 (2.2, 4.6)**	**1.2 (0.1, 2.3)**	**5.8 (2.4, 9.2)**	3.0 (-0.2, 6.3)
20 + persons	**2.0 (0.6, 3.3)**	0.9 (-0.4, 2.1)	3.7 (-0.2, 7.7)	2.3 (-1.3, 5.9)
Pet (ref: Yes)				
No	0.6 (-0.1, 1.3)	0.1 (-0.5, 0.8)	**3.7 (1.7, 5.8)**	0.6 (-1.4, 2.6)
Number of chronic conditions (ref: None)				
One	-0.4 (-1.2, 0.5)	-0.02 (-0.8, 0.7)	**-4.5 (-6.9, -2.1)**	-1.6 (-3.8, 0.6)
Two	**-2.1 (-3.1, -1.2)**	-0.8 (-1.7, 0.1)	**-9.6 (-12.4, -6.8)**	**-2.8 (-5.5, -0.1)**
Three or more	**-5.4 (-6.6, -4.1)**	**-2.6 (-3.8, -1.3)**	**-17.6 (-21.1, -14.0)**	**-7.5 (-11.3, -3.8)**
COVID1: I have lost touch with many people since the COVID-19 (ref: Strongly Disagree)				
Somewhat Disagree	**-3.3 (-4.3, -2.3)**	-0.3 (-1.5, 0.8)	**-9.0 (-11.9, -6.0)**	1.0 (-2.4, 4.4)
Do not Agree nor Disagree	**-7.1 (-8.2, -5.9)**	-0.9 (-2.2, 0.4)	**-14.8 (-18.1, -11.4)**	1.9 (-2.0, 5.8)
Somewhat Agree	**-5.2 (-6.1, -4.3)**	-0.01 (-1.1, 1.1)	**-18.8 (-21.5, -16.1)**	0.6 (-2.8, 4.0)
Strongly Agree	**-9.6 (-10.9, -8.4)**	-0.9 (-2.5, 0.6)	**-30.2 (-33.6, -26.7)**	-1.8 (-6.5, 2.9)
COVID2: It takes a lot of energy to connect with friends during the COVID-19 pandemic (ref: Strongly Disagree)				
Somewhat Disagree	**-2.6 (-3.7, -1.5)**	0.1 (-1.1, 1.3)	**-8.4 (-11.6, -5.3)**	**-3.6 (-7.2, -0.03)**
Do not Agree nor Disagree	**-6.4 (-7.5, -5.3)**	-0.3 (-1.5, 1.0)	**-16.2 (-19.3, -13.0)**	-1.4 (-5.2, 2.4)
Somewhat Agree	**-5.0 (-5.9, -4.0)**	0.2 (-0.9, 1.4)	**-19.3 (-22.1, -16.6)**	-3.3 (-6.8, 0.1)
Strongly Agree	**-9.1 (-10.4, -7.9)**	-0.01 (-1.5, 1.5)	**-33.6 (-37.0, -30.1)**	-4.5 (-9.0, 0.02)
COVID3: The increased social isolation from the COVID-19 pandemic has had a significant negative impact on my mental health (ref: Strongly Disagree)				
Somewhat Disagree	**-3.6 (-4.5, -2.7)**	**-1.6 (-2.7, -0.5)**	**-10.2 (-12.6, -7.8)**	-2.5 (-5.7, 0.8)
Do not Agree nor Disagree	**-7.2 (-8.1, -6.3)**	**-1.5 (-2.8, -0.2)**	**-20.4 (-22.9, -17.9)**	**-6.0 (-9.9, -2.1)**
Somewhat Agree	**-8.2 (-9.1, -7.3)**	**-1.8 (-3.2, -0.5)**	**-28.8 (-31.1, -26.4)**	**-7.6 (-11.4, -3.7)**
Strongly Agree	**-13.0 (-14.3, -11.7)**	**-2.7 (-4.8, -0.7)**	**-43.9 (-47.3, -40.4)**	**-12.6 (-18.6, -6.7)**
COVID4: The increased social isolation from the COVID-19 pandemic has had a significant negative impact on my physical health (ref: Strongly Disagree)				
Somewhat Disagree	**-4.2 (-5.1, -3.4)**	-0.8 (-1.9, 0.3)	**-10.0 (-12.5, -7.5)**	-0.9 (-4.2, 2.3)
Do not Agree nor Disagree	**-7.8 (-8.7, -6.9)**	**-1.4 (-2.6, -0.1)**	**-20.5 (-23.2, -17.9)**	-2.0 (-5.7, 1.8)
Somewhat Agree	**-6.9 (-7.8, -6.0)**	-1.0 (-2.1, 0.2)	**-25.1 (-27.6, -22.6)**	-2.8 (-6.3, 0.6)
Strongly Agree	**-12.5 (-13.8, -11.2)**	-1.4 (-3.3, 0.5)	**-37.9 (-41.6, -34.1)**	1.2 (-4.5, 6.8)
COVID5: The COVID-19 pandemic has caused my stress and/or anxiety levels to increase (ref: Strongly Disagree)				
Somewhat Disagree	**-3.7 (-4.7, -2.7)**	**-1.4 (-2.6, -0.2)**	**-9.5 (-12.1, -6.8)**	**-4.0 (-7.5, -0.6)**
Do not Agree nor Disagree	**-6.8 (-7.9, -5.8)**	**-1.5 (-2.8, -0.2)**	**-17.2 (-20.0, -14.5)**	**-4.7 (-8.5, -1.0)**
Somewhat Agree	**-5.7 (-6.5, -4.9)**	-0.9 (-2.0, 0.2)	**-23.2 (-25.4, -21.0)**	**-10.1 (-13.3, -6.9)**
Strongly Agree	**-12.3 (-13.5, -11.2)**	**-1.8 (-3.4, -0.2)**	**-43.9 (-46.8, -40.9)**	**-18.6 (-23.3, -13.8)**
**Scales**				
Social Network (Lubben)	**0.6 (0.6, 0.7)**	**0.2 (0.1, 0.2)**	**1.3 (1.2, 1.5)**	0.1 (-0.1, 0.3)
Loneliness (DJ)	**-2.7 (-2.8, -2.5)**	**-1.4 (-1.6, -1.2)**	**-7.2 (-7.6, -6.7)**	**-3.7 (-4.3, -3.0)**

*Results with a p-value < 0.05 are in bold face

† The adjusted model includes all variables listed in the table

95% CI = 95% confidence interval

Figures 2 and 3 illustrate that the two COVID-19 items related to mental health also demonstrated near-stepwise associations with reduced QoL. Compared to those who strongly disagreed that COVID-19 had a negative impact on their mental health, those who strongly agreed experienced a lower QoL (β= -2.7, 95% C.I. =-4.8,-0.70). Even those who somewhat disagreed with this statement were more likely to have reduced QoL. Similarly, those who strongly agreed that the pandemic has caused their stress/anxiety to increase experienced lower QoL. Having social contact with 10–19 people was associated with higher QoL, while this was not seen for those having social contact with 5–9 or more than 20 people, compared to 0–4 contacts.

Respondents with three or more chronic conditions had a significantly lower QoL (β=-2.6, 95% C.I. = -3.8, -1.3) than those reporting no chronic conditions. Lonelier respondents, as measured by the DeJong scale, (β=-1.4, 95% C.I. = -1.6, − 1.2) were more likely to have a lower QoL, although the association between social engagement and QoL was very weak (β = 0.2, 95% C.I.= 0.1,0.2). Compared to Caucasians, Asian/Pacific Islanders were less likely to have a higher quality of life.

In terms of well-being, a somewhat different pattern of associations was evident. The two COVID-19 questions related to mental health were again found to have significant stepwise associations with well-being. Compared to Caucasians and Asian/Pacific Islanders, those in the “other” category reported better well-being. There was a weak positive association between a tenure at their current residence of 10 years or more and well-being. The sole relationship between well-being and income was a positive association for those who did not disclose their status, while there was no difference in well-being between those whose income always met their needs and those whose income never met their needs. Reporting either two or three or more chronic conditions were related to reduced well-being. No association between social isolation and well-being was found, and the relationship between loneliness and well-being was weak (β=-0.09,95% C.I. = -1.1, -0.8).

### Sex differences

The presence of chronic conditions and loneliness were associated with QoL and well-being for both females and males. Income was associated with QoL for both sexes, but was associated only with well-being for females whose needs were met all of the time or who did not disclose income status. The influence of other factors, such as region of residence, ethnicity, stresses related to COVID, and social contact, on QoL and well-being was complex. Associations between QoL and ethnicity, region of residence, and social contact were noted among females, but not males. Social engagement was associated with QoL in both sexes, whereas social engagement was related to well-being only for males who had social contact with 20 or more people.

The relationship between well-being and increased stress as a result of COVID were similar for females and males. While there were differences between males and females in the associations between the impact of COVID variables (negative impact on mental health, negative impact on physical health, and increased stress/anxiety) and QoL, interaction assessment showed that there was only statistically significant interaction between sex and income and sex and social contact.

## Discussion

### Main findings

This study investigated predictors of QoL and well-being among older Canadian adults within the context of the pandemic, including loneliness and social isolation.

The mean score of 53.7 (S.D.=8.2) out of a total of 65 possible points on the OPQoL-B essentially replicated scores on the same instrument from pre-pandemic surveys of Australian and Persian older adults [42, 50]. Longitudinal studies of older adults have reported that QoL has remained relatively stable before and during the pandemic [51, 52]. Positive associations were noted in the present study between QoL and: the ability of income to meet needs; higher levels of education; daily social contact with 10–19 people; and social engagement. Reporting three or more chronic conditions, higher levels of loneliness, agreeing that the pandemic had a negative effect on mental health, that the pandemic had caused stress and/or anxiety levels to increase, or claiming ethnicity as an Asian/Pacific Islander were factors associated with a decreased QoL.

In comparison to other surveys examining QoL of older adults during the pandemic, [53–55] we found similar associations between QoL and financial circumstances and between QoL and education levels. In our study, the only association between QoL and chronic conditions was found for those respondents reporting more than three conditions, whereas two of the other studies reported associations between the absence or presence of chronic conditions and QoL [53, 54]. However, we did not find the associations reported in these studies between QoL and age, marital status, or living situation. The differences in factors associated with QoL between the studies may reflect variability in living conditions and available supports between Canada and the other surveys, which had been conducted in Asia, China, and Iran, [53–55] as well as the measures used to assess QoL.

The mean well-being score of 59.6 (S.D.=6.0) among our respondents is considered “good” [56]. These scores can be contextualized in reference to WHO-5 scores of 14,975 adults in the multinational Activity and Health during Sars-CoV2 Pandemic (ASAP) study, which declined from 68.1 ± 16.9 to 51.9 ± 21 during COVID restrictions [57]. Several longitudinal studies, however, have reported that well-being among older adults was unaltered, [58] or even improved, [59, 60] during the pandemic.

In the present study, claiming an ethnicity other than Caucasian or Asian/Pacific Islander, residing in the province of Quebec, declining to report whether income met needs, or residing in the same residence for 10 years or more were associated with higher levels of well-being. Negative relationships were noted between well-being and: being female; having two or more chronic conditions; being neutral or agreeing that the pandemic had a significant impact on mental health or that it caused an increase in stress and anxiety; or reporting more loneliness. While no differences in QoL between sexes were found in this study, a large study of older adults in low- and middle- income countries found that males reported a better QoL than females [61]. This discrepancy may be the result of differences in life expectancies, health care systems, and levels of social and economic development [61].

The COVID-19 pandemic and the concomitant public health restrictions increased stress and/or anxiety levels in more than 40% of respondents in this study, with over one-quarter reporting a negative effect on their mental health. Data from the Canadian Longitudinal Study on Aging COVID-19 exit questionnaire, [62] conducted earlier in the pandemic (September-December, 2020), found that 75.5% of older adults experienced at least one stressor during this time frame, with 63.6% perceiving the consequences as negative. This proportion is within the broad range of 23.2% [63] -84.5% [64] of older adults who reported anxiety during the pandemic.

Despite the increased risk for poor health outcomes during the COVID-19 pandemic, studies across multiple Western nations have reported that older adults experience fewer mental health effects than younger persons [63, 65–69]. This phenomenon has been attributed to lower stress reactivity and better emotional regulation and resilience among older adults [70]. Older adults were found to perceive the risks associated with COVID-19 to be higher than younger adults, although older men were less worried than younger men [71].

It has been noted that QoL, which captures cognitive judgements, and well-being, which addresses the emotional evaluations, show different associations with individual characteristics and life circumstances, [16] which was the case in the present study. The ability of income to meet needs demonstrated strong associations with increased QoL, supporting previous studies examining the relationships between these factors in older adults [72–74]. This study did not find a corresponding relationship, however, between the ability of income to meet needs and well-being. A significant positive relationship with well-being was found only for those who declined to disclose their response to the ability of income to meet needs.

While loneliness was associated with both poorer QoL and well-being in the present study, the relationships were relatively weak compared to other covariates. Findings from other studies regarding these relationships have been mixed, partly as result of the measures used as well as the cultural and social contexts of the studies. While some studies found a clear relationship between loneliness and QoL, [75–78] Beridze et al. [31] reported that loneliness was associated with QoL in Swedish older adults, but that association was limited to higher levels of loneliness. In Spain, however, loneliness was not significantly associated with QoL in older adults [31].

For older adults, social isolation may result due to relationship losses, impaired health, and/or changes in living arrangements that may have been compounded by COVID-19 restrictions Social isolation was weakly associated with QoL, but not associated with well-being in the present study. While previous studies have reported that social isolation in older adults is associated with decrements in QoL, [79–81] the unique circumstances of social isolation due to the pandemic may have altered respondents’ expectations about the frequency and availability of social support.

### Strengths and Limitations

This study has several important strengths. The use of an anonymous, web-based survey during the COVID-19 pandemic increased accessibility to respondents across the country and limited participant burden. Older males, who are often under-represented in studies of seniors, comprised 44.3% of our sample. Sex differences in QoL and well-being were described, revealing the complex nature of these associations. In addition, diversity in socioeconomic status was reflected in the 898 older adults (40.7%) who reported that their incomes met their needs either none of the time or only some of the time. Given the wealth of instruments measuring QoL, the use of the OPQoL-B which had been specifically designed for and validated in older adults, ensured relevance to age-specific QoL. Taken together, these strengths have yielded results that describe the experience of Canadian older adults during the COVID-19 pandemic.

Limitations of the study include the generalizability of the findings beyond older adults residing in the community who had access and skills to the internet through phones or computers to have been reached by the survey recruitment protocol. Limitations to the current study also include selection bias as online surveys are restricted to those who have internet access with technology literacy. Studies have found internet use among older adults could promote well-being, reduce loneliness, and drive social engagement [82, 83]. Internet and technology use are also associated with better health status [84].

Ethnic diversity of the sample was limited. The cross-sectional design of this study did not allow us to examine changes in QoL or well-being over time, and limited interpretations about causality. Data collection occurred over several months during the rapidly changing circumstances associated with the COVID-19 pandemic and public restrictions, which may have affected responses to the survey.

## Conclusions

The COVID-19 pandemic is associated with differential effects between sub-groups of older adults. In particular, those with limited financial resources and those with multiple chronic conditions may be at more risk to suffer adverse QoL and well-being consequences. Additional research is needed to identify the role ethnicity may play in older adults’ appraisals of QoL and well-being. Income support for older adults living in relative disadvantage is likely to mitigate some of the negative impacts on QoL and well-being, regardless of whether societal pressures such as a pandemic are occurring. As one of the predictors of decreased QoL and well-being, interventions designed to address the potentially modifiable risk factor of loneliness among older adults may enhance these important outcomes.

## Data Availability

The datasets used and/or analyzed are available from the corresponding author on reasonable request.

## Abbreviations

CHASR: Canadian Hub for Applied and Social Research
DjG: DeJong Gierveld Loneliness Scale
LSNS-6: Lubben Social Network Scale
OPQoL: Older People’s Quality of Life Scale
OPQoL-B: Older People’s Quality of Life Scale - Brief
QOL: Quality of life
